# Effects of Transcranial and Trans-Spinal Direct Current Stimulation Combined with Robot-Assisted Gait Training on Gait and Fatigue in Patients with Multiple Sclerosis: A Double-Blind, Randomized, Sham-Controlled Study

**DOI:** 10.3390/jcm13247632

**Published:** 2024-12-14

**Authors:** Gülser Cinbaz, Zübeyir Sarı, Semra Oğuz, Temel Tombul, Lütfü Hanoğlu, Juan J. Fernández-Pérez, Julio Gómez-Soriano

**Affiliations:** 1 Faculty of Health Sciences, Istanbul Medeniyet University, 34862 Istanbul, Turkey; 2 Faculty of Health Sciences, Marmara University, 34854 Istanbul, Turkey; zubeyirsari@gmail.com (Z.S.); semra.oguz@marmara.edu.tr (S.O.); 3 Department of Neurology, Faculty of Medicine, Istanbul Medeniyet University, 34720 Istanbul, Turkey; temeltombul@yahoo.com; 4 Department of Neurology, Faculty of Medicine, Istanbul Medipol University, 34810 Istanbul, Turkey; lhanoglu@kure.com.tr; 5 Toledo Physiotherapy Research Group (GIFTO), Faculty of Physiotherapy and Nursing of Toledo, Universidad de Castilla-La Mancha, 45004 Toledo, Spain; juanjose.fernandez@uclm.es (J.J.F.-P.); julio.soriano@uclm.es (J.G.-S.); 6 Instituto de Investigación Sanitaria de Castilla-La Mancha (IDISCAM), 45004 Toledo, Spain

**Keywords:** multiple sclerosis, transcranial direct current stimulation, trans-spinal direct current stimulation, neurological rehabilitation, gait

## Abstract

**Background/Objectives:** Multiple Sclerosis (MS) is a chronic neurological condition that impairs motor and sensory functions, particularly gait. Non-invasive neuromodulation techniques aim to enhance functional recovery and motor–cognitive outcomes, though their effectiveness remains debated. This study compared the effects of transcranial direct current stimulation (tDCS) and trans-spinal direct current stimulation (tsDCS), combined with robotic-assisted gait training (RAGT), on motor function and fatigue in people with MS (pwMS). **Methods:** This double-blind, randomized, sham-controlled clinical trial included 35 pwMS, who participated in 12 sessions of 20 min anodal tDCS (n = 11), cathodal tsDCS (n = 12), or sham treatment (n = 12), in addition to RAGT. Primary outcomes were assessed using the Timed 25-foot Walk (T25-FW), Timed Up and Go (TUG), walking speed, and Multiple Sclerosis Walking Scale-12 (MSWS-12). Fatigue was assessed with the Fatigue Severity Scale (FSS) and the Fatigue Impact Scale (FIS). ClinicalTrials number: NCT06121635. **Results:** Significant improvements in gait speed, T25-FW, MSWS-12, TUG scores, and fatigue (FSS) favored tDCS and tsDCS over sham stimulation. While no differences were found between tDCS and tsDCS, the tsDCS group showed a significant improvement in the FIS physical subscale compared to sham, unlike the tDCS group. **Conclusions:** tDCS and tsDCS, combined with RAGT, improve walking and reduce fatigue in pwMS, highlighting their potential in motor rehabilitation.

## 1. Introduction

Multiple Sclerosis (MS) is a chronic neurological condition characterized by inflammation, demyelination, and neurodegeneration within the central nervous system [1]. The wide-ranging symptoms of MS, such as muscle weakness, sensory disturbances, impaired coordination, and cognitive deficits, can vary significantly among individuals [2]. Impaired mobility, especially difficulties with gait, is among the most debilitating and frequently observed symptoms [3]. Motor and sensory impairments resulting from central nervous system dysfunction lead to disturbances in gait and balance. These issues compromise postural control in people with MS (pwMS), causing postural instability that negatively affects walking patterns [4]. To counteract this instability, pwMS often adopt a more cautious gait. Although this adjustment may offer temporary stability, it often results in inefficient walking patterns, increased energy expenditure, and muscle fatigue, further compounding mobility issues and increasing the likelihood of falls [5].

Emerging therapeutic interventions, including robotic-assisted gait training (RAGT), neuromodulation, and tailored rehabilitation programs, offer promising avenues for enhancing motor function, improving balance, and mitigating the impact of disease progression on mobility [6]. In recent years, non-invasive neuromodulation techniques have emerged as complementary approaches aimed at accelerating functional recovery and enhancing motor and cognitive outcomes [7]. Techniques such as transcranial direct current stimulation (tDCS) and trans-spinal direct current stimulation (tsDCS) are favored for their safety, tolerability, affordability, portability, and ease of use [7,8]. However, the effectiveness of these techniques remains controversial [9,10].

Transcranial direct current stimulation (tDCS) modulates neural activity and enhances therapeutic outcomes by applying low-intensity electrical currents to targeted brain regions via electrodes placed on the scalp [11,12]. Lefaucheur et al. [13] reviewed the impact of tDCS on the central nervous system, noting that anodal tDCS stimulation of the contralateral primary motor cortex (M1) enhances corticospinal tract output. This increase in strength and motor-evoked potentials (MEP) may contribute to improved motor function in both upper and lower limbs, making tDCS a valuable tool in motor rehabilitation for patients with neurological conditions [14].

Nonetheless, the available evidence on the efficacy of tDCS in pwMS remains inconclusive. A systematic review and meta-analysis [10] assessing the impact of tDCS on gait and balance in pwMS revealed significant improvements in gait functionality measures. However, no significant differences were observed in static balance outcomes or patients’ self-perception of their gait abilities [10]. Additionally, the heterogeneity in locations and parameters used makes it difficult to select a specific protocol for clinical application.

Trans-spinal direct current stimulation (tsDCS) involves the delivery of continuous electrical currents via electrodes positioned at various spinal cord levels [15]. While the underlying neurophysiological mechanisms are still under investigation, some researchers suggest that tsDCS may alter neuronal membrane potentials, influencing spinal networks and cortical connectivity [15]. Research on healthy participants has shown that anodal tsDCS can decrease the nociceptive flexor reflex (NFR), while cathodal tsDCS has been associated with both an increase in NFR and a rise in MEP amplitude [16,17].

Various studies have shown varied effects of tsDCS on motor functions in individuals with neurological disorders. A systematic review [9] examined the effectiveness of tsDCS in this population. Specifically, Lamy et al. [18] found that participants in the cathodal tsDCS group exhibited a significant increase in time spent standing during a 60 min session compared to those in the sham group. However, two other studies [19,20] included in the review reported no significant changes in H-reflex or MEP amplitudes following cathodal stimulation. Additionally, a study [21] involving a participant with spinal cord injury demonstrated that combining tsDCS with RAGT reported improvements in motor function, walking parameters, and cortical excitability.

Given this context, tDCS and tsDCS combined with neurorehabilitation may provide additional benefits to walking parameters in pwMS. Accordingly, the primary aim of this study was to compare the effects of tDCS and tsDCS, administered alongside RAGT, on motor function in pwMS. Moreover, the effects of tDCS and tsDCS were compared to sham stimulation to determine each intervention’s efficacy. A secondary aim was to explore the relationship between these neuromodulation techniques and fatigue through comprehensive fatigue assessments.

## 2. Materials and Methods

### 2.1. Study Design

This study was a double-blind, randomized, sham-controlled clinical trial in pwMS. Prior to baseline evaluation, an independent researcher used the website https://www.randomizer.org/ (accessed on 11 December 2023) to randomly allocate participants into three distinct groups: the anodal tDCS group, the cathodal tsDCS group, and the sham group. To maintain participant blinding, pwMS were not informed of their group assignment, and a sham protocol was implemented. Physiotherapists administering the exercise program and assessments were also blinded to the participants’ stimulation group.

This study received ethical approval from the Istanbul Marmara University School of Medicine’s ethics committee (ID number: 09.2020.1075), adhering to the ethical principles for medical research involving human subjects as outlined in the Declaration of Helsinki. Prior to participation, all participants were informed about the study procedures and provided written informed consent. The study was registered with the ClinicalTrials.gov Protocol Registry and Results System (registration number: NCT06121635).

### 2.2. Participants and Settings

The sample was recruited at the Neurology Polyclinic of Istanbul Prof. Dr. Süleyman Yalçın City Hospital (Türkiye). A neurologist assessed participants for eligibility and referred those meeting the inclusion criteria to the study. Assessments and treatment programs were conducted at the Neurological Rehabilitation Unit of the Istanbul Cadde Medical Center (Türkiye). The inclusion criteria were as follows: (a) MS diagnosis according to the McDonald criteria [22], (b) an Expanded Disability Status Scale (EDSS) score between 2 and 6, and (c) the ability to walk at least 25 m with or without assistance. The exclusion criteria included the following: (a) a relapse within the last 2 months, (b) medication change within the last 45 days, (c) hospitalization within the last 90 days, (d) the presence of other neurological or musculoskeletal pathology, (e) skin conditions preventing electrical stimulation (sensitivity, open wound, etc.), (f) the use of medications containing fampridine, and (g) receipt of botulinum toxin treatment within the last 6 months.

The sample size was calculated using G*Power 3.1.9.4 software. To ensure 80% statistical power, we based our sample size on repeated measures and within-group interaction analyses as primary statistical tests, resulting in a required sample size of 12 participants per group, totaling 36 participants.

### 2.3. Interventions

Participants completed a 4-week physiotherapy program 3 times per week, totaling 12 sessions with 1-day intervals between each session. Each session consisted of 20 min of electrical stimulation (offline mode), 20 min of robot-assisted gait training, and 15 min of balance and gait training. Participants were randomly assigned to one of three stimulation groups as follows: (1) anodal tDCS + sham tsDCS, (2) cathodal tsDCS + sham tDCS, or (3) sham tDCS + sham tsDCS. Cranial and spinal electrodes were applied to all participants. In the anodal tDCS group, cranial electrodes were active, while spinal electrodes remained in sham mode. In the cathodal tsDCS group, spinal electrodes were in active mode, while cranial electrodes were in sham mode. In the sham group, both cranial and spinal electrodes were set to sham mode. The balance and gait training comprised various exercises [23], including strengthening exercises, stair climbing, balance board training, soft floor exercises, and Bosu exercises. A detailed explanation of the exercise training program and dosage is provided in Table 1.

Participants received two types of direct current stimulation simultaneously in offline mode, administered for 20 min per session across 12 sessions. Participants were seated in a comfortable, supported position during stimulation [24].

#### 2.3.1. Transcranial Direct Current Stimulation (tDCS)

tDCS stimulation was delivered using a direct current stimulator (TCT Research Limited, Hong Kong) connected to a pair of rectangular electrodes (7 × 5 cm^2^) rubber-wrapped with a sponge soaked in saline solution (0.9% NaCl) [24,25].

Electrode placement followed the International EEG 10/20 System. The active electrode (anode) was positioned over the left M1 (C3), while the reference electrode (cathode) was placed contralaterally on the supraorbital region [13,26]. To locate M1, the distance between the nasal root (nasion) and the occipital protuberance (inion) was measured, with the midpoint marking Cz. In addition, the distance between the tragus was measured and recorded. Then, the M1 point was determined by moving laterally by 20% of the recorded distance from the vertex [13]. The electrodes were fixed with customized elastic strips.

Electrodes were secured using custom elastic straps. In the anodal tDCS group, a direct current of 2 mA with a density of 0.057 mA/cm² was applied. Conversely, in the sham tDCS group, the current was reduced to 0 mA following the initial 30 s of stimulation.

In this study, anodal tDCS was selected due to its well-documented facilitative effects on cortical excitability. Anodal tDCS stimulation has been shown to depolarize neuronal membranes, thereby lowering the threshold for action potential generation and enhancing synaptic activity in the targeted cortical region [11,12]. This mechanism is particularly advantageous for motor rehabilitation, as it supports neuroplasticity and promotes the reorganization of motor pathways, which are critical for improving motor functions [13,14].

#### 2.3.2. Trans-Spinal Direct Current Stimulation (tsDCS)

tsDCS was administered using a direct current stimulator (TCT Research Limited, Hong Kong) paired with rectangular electrodes (7 × 5 cm^2^) made of rubber and covered with saline solution-soaked sponges (0.9% NaCl) [24,25].

The active electrode (cathode) was placed over the T10 spinous process, while the reference electrode (anode) was positioned horizontally on the left posterior deltoid [24]. Electrodes were secured with custom elastic straps. A direct current of 2 mA with a density of 0.057 mA/cm² was applied to the cathodal tsDCS group. In the sham tsDCS group, the current was subsequently reduced to 0 mA after the initial 30 s of stimulation.

In this study, cathodal tsDCS was chosen due to its capacity to modulate spinal excitability and its well-documented effects on spinal networks and cortical connectivity [15]. Research has demonstrated that cathodal tsDCS directly influences neuronal membrane potentials, leading to increased motor-evoked potential (MEP) amplitudes and significant alterations in spinal reflex pathways [16,17]. Specifically, cathodal tsDCS has been shown to increase the nociceptive flexor reflex (NFR), thereby enhancing motor output through spinal mechanisms. Conversely, anodal tsDCS has been shown to decrease the NFR, resulting in reduced motor output and lowered excitability in spinal circuits, making cathodal stimulation more suitable for the aim of this study [16,17].

#### 2.3.3. Robot-Assisted Gait Training (RAGT)

Robot-assisted gait training was performed using the LokoHelp Woodway gait robot. This device, mounted on a motorized treadmill frame, simulates the gait cycle by transmitting the treadmill’s movement to levers on either side, replicating stance and swing phases accurately. Participants were secured in a body-weight support harness above the LokoHelp [27]. The gait training was conducted at a speed of 1.5 km/h for the first 10 min, followed by 2.0 km/h for the remaining 10 min, with 20% body weight supported.

### 2.4. Outcomes

Participants were assessed both at baseline (pre-treatment) and after a 4-week treatment program (post-treatment). The main outcomes included the Timed 25-foot Walk test (T25-FW), the Timed Up and Go test (TUG), gait speed, and scores on the Multiple Sclerosis Walking Scale-12 (MSWS-12).

The Timed 25-foot Walk (T25-FW) was used to measure gait function. Participants were asked to walk a marked 25-foot course in a straight line as quickly and safely as possible, with or without a walking aid. The total time was recorded in seconds. This measurement scale has been validated and has shown excellent reliability in pwMS [28].

The TUG test was used to measure dynamic balance. Participants were instructed to stand up from a chair, walk 3 m at their own pace, make a 180° turn around a cone, and then sit back down. The time taken for the entire process was recorded in seconds. This measure was reported to be valid in pwMS [29].

Regarding gait speed, participants were asked to walk at their normal pace in a standardized 10 m environment, and the total time was recorded. Gait speed was calculated by dividing the distance walked by the recorded time [28].

The Multiple Sclerosis Walking Scale-12 (MSWS-12) is a 12-item functional outcome measure used to assess individuals’ perspectives on how MS affects their walking ability. Participants rated the impact of movements, such as standing, walking, running, and climbing stairs on a 5-point Likert scale (1 = almost not at all, 5 = extremely). Higher scores indicate a greater negative impact on walking ability [30]. This scale has been adapted and validated for the Turkish population and has shown good reliability in pwMS [31].

The secondary outcomes included the Fatigue Severity Scale (FSS) and the Fatigue Impact Scale (FIS). The FSS is a Likert-type scale (0–7) comprising 9 questions that assess experiences, causes, and effects of fatigue, with scores of 36 or above indicating a high perception of fatigue severity [32]. The FIS evaluates the impact of fatigue on daily activities, assessing physical, cognitive, and social effects over the past month with 40 questions (from 0 = no problem to 4 = extreme). The total score ranges from 0 to 160, with higher scores indicating a greater impact of fatigue [33]. Given that our study focused on changes in walking, a physical parameter, we also included the physical subscale of the FIS in the outcomes. Both scales have been adapted and validated for the Turkish population, demonstrating good reliability for assessing fatigue in pwMS [32,33].

### 2.5. Statistical Analysis

Statistical analyses were performed using SPSS 23.0 software (SPSS Inc., Chicago, IL, USA). Non-parametric tests were used due to the small sample sizes in the groups. Descriptive analyses were given as percentages for categorical variables, while median and minimum–maximum values were given for ordinal and numerical variables. Initial values between groups were compared using the Kruskal–Wallis test for numerical variables and the Pearson Chi-Square test for categorical variables. The Wilcoxon signed-rank test was used for within-group comparisons. The Kruskal–Wallis test was used to calculate differences between groups, with delta values (pre–post differences within each group) serving as the basis for comparison. Bonferroni correction was applied to identify which group/groups contributed to the observed differences between groups. A *p*-value of <0.05 was considered statistically significant.

## 3. Results

**Figure 1:**
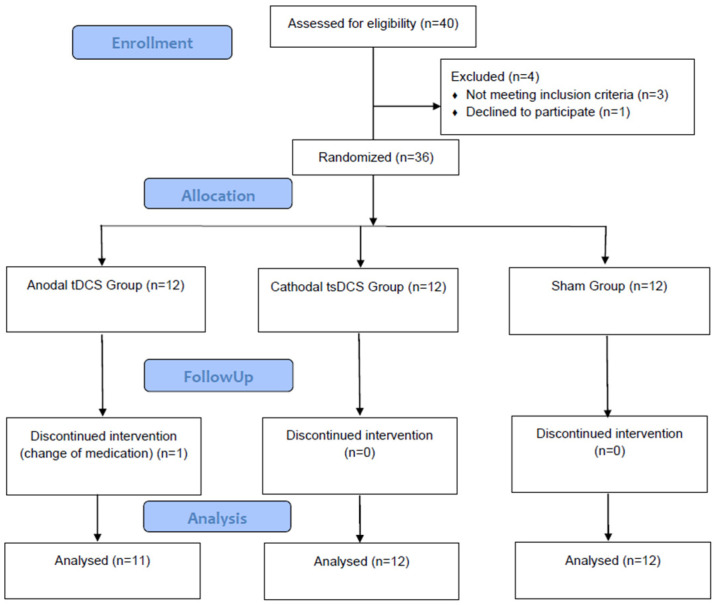
Study flow diagram.

From December 2023 to May 2024, a total of 40 people with Multiple Sclerosis (pwMS) were screened for eligibility. Of these, three were excluded due to not meeting the inclusion criteria, and one declined participation due to transportation issues. Consequently, 36 participants were randomized. However, one participant in the anodal tDCS group was excluded post-randomization due to a change in medication regimen. Finally, 35 participants completed all sessions and were included in the final analysis. The study flow diagram is presented in Figure 1.

The median age and disease duration were similar across the three groups. In the anodal tDCS group, the median age was 49.0 (36.0–71.0) years, with a disease duration of 16.0 (7.0–24.0) years. Participants in the cathodal tsDCS group had a median age of 49.0 (30.0–75.0) years and a median disease duration of 13.5 (4.0–36.0) years. In the sham group, the median age was 48.5 (37.0–66.0) years, while the median disease duration was 20.0 (2.0–37.0) years. Regarding MS type distribution, the RRMS type was the most prevalent in all groups (63.6%, 58.3%, 66.7%). The mean EDSS scores were 4.5 (3.5–5.5) in the anodal tDCS group, 5.0 (3.5–6.0) in the cathodal tsDCS group, and 3.7 (3.0–6.0) in the sham group. Detailed information about the demographic characteristics and baseline clinical outcomes of the participants can be found in Table 2. No statistically significant differences in baseline parameters were detected between groups. Both types of interventions were well tolerated across groups, and no adverse effects were observed in participants.

### 3.1. Gait Function

Within-group analyses demonstrated significant improvements in T25-FW (*p* = 0.003), MSWS-12 (*p* = 0.003), and TUG scores (*p* = 0.003) for the anodal tDCS group, as well as a notable increase in walking speed (*p* = 0.003) (Table 3). Similarly, the cathodal tsDCS group also exhibited significant improvements in T25-FW (*p* = 0.002), MSWS-12 (*p* = 0.002), and TUG scores (*p* = 0.002), along with an increase in walking speed (*p* = 0.002) (Table 3). Conversely, the sham group exhibited no significant changes in motor function variables related to walking (*p* > 0.05).

**Figure 2:**
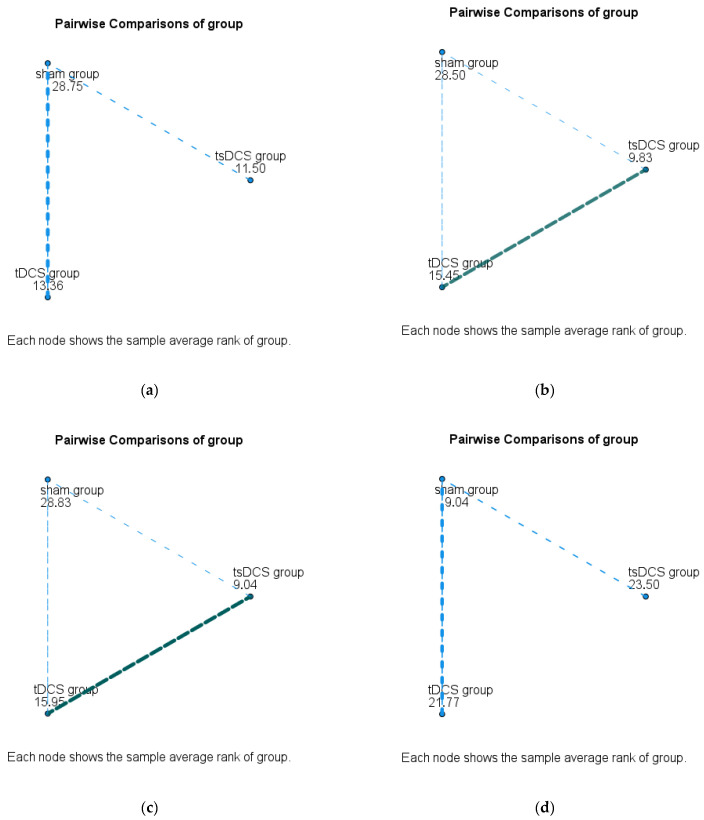
Pairwise comparisons indicating the source of the difference between groups. (**a**) Pairwise comparisons for T25FW; (**b**) pairwise comparisons for TUG; (**c**) pairwise comparisons for MSWS-12; (**d**) pairwise comparisons for gait speed.

Between-group comparisons indicated statistically significant differences in T25-FW, TUG, walking speed, and MSWS-12 scores (*p* < 0.001) (Table 3). Post hoc analyses identified significant improvements in T25-FW, gait speed, MSWS-12, and TUG scores for the tDCS group compared with the sham group (*p* < 0.05). Similarly, the tsDCS group showed significant improvements in T25-FW, gait speed, MSWS-12, and TUG scores compared with the sham group (*p* < 0.05). However, no significant differences were observed between the tDCS and tsDCS groups (*p* > 0.05) (Table 3 and Figure 2).

### 3.2. Fatigue

Within-group comparisons revealed significant decreases in the FSS and FIS-physical scores in the tDCS group (*p* < 0.05). Similarly, the tsDCS group also demonstrated significant decreases in FSS and FIS-physical scores (*p* < 0.05). However, the sham group showed no significant changes in these scores (*p* > 0.05).

When analyzing the FIS-total scores, statistically significant improvements were observed solely in the tsDCS group (*p* = 0.041), whereas no significant differences were noted in the tDCS group (*p* = 0.170) or the sham group (*p* = 0.449) (Table 3).

In inter-group comparisons, significant differences were found for the FSS and FIS-physical scores (*p* < 0.05). However, no significant differences were observed for the FIS-total scores (*p* > 0.05) (Table 3). Post hoc analysis indicated significant improvements in FSS scores for the tDCS group compared with the sham group (*p* = 0.032). Similarly, significant improvements in FSS scores were found for the tsDCS group compared with the sham group (*p* = 0.004). For FIS-Physical outcomes, significant differences were detected only in the tsDCS group compared with the sham group (*p* = 0.047). No significant differences were found between the tDCS and tsDCS groups for any of the outcomes (Table 3).

## 4. Discussion

This randomized controlled trial is the first to compare the effects of cathodal tsDCS and anodal tDCS versus sham stimulation on gait function and fatigue in pwMS. The results revealed significant improvements in gait-related outcomes, including increased gait speed and reductions in T25-FW, MSWS-12, and TUG scores, as well as decreased fatigue (measured by FSS), favoring both tDCS and tsDCS over sham stimulation. However, no differences were observed between the tDCS and tsDCS groups across any measured outcomes. Particularly, the tsDCS group demonstrated a significant improvement in the physical subscale of the FIS compared to the sham group, which was not observed in the tDCS group.

The mechanisms underlying the observed outcomes in the tDCS group may involve the modulation of neuronal plasticity processes. Previous studies using magnetic resonance imaging (MRI) have shown that focal lesions, or plaques, commonly present in MS can alter cortical excitability, thereby contributing to MS symptoms [4]. As a result, regulating cortical excitability has emerged as a potential therapeutic approach. By enhancing excitability in the M1, tDCS may stimulate neural circuits that support neuroplastic processes across networks essential for gait and central nervous system control centers, such as the cerebral cortex, cerebellum, and basal ganglia [13,34]. MRI findings indicate that tDCS increases connectivity between previously inactive brain regions, suggesting the activation of new or previously dormant neural networks [35]. Thus, tDCS may facilitate brain network reorganization, modifying neuronal activity through the regulation of cortical excitability.

A recent systematic review and meta-analysis [10] examining the effects of tDCS on gait and balance in pwMS highlighted significant improvements across multiple aspects of gait, consistent with our findings. Specifically, the meta-analysis reported significant improvements in widely used clinical assessments, including the TUG, 25FWT, and 2-minute Walk Test (2MWT), reflecting enhanced motor performance and functional mobility [10]. While our study noted improvement in self-reported walking ability, measured by the MSWS-12, the meta-analysis did not observe significant changes in this subjective measure. This discrepancy may stem from variations in study design, participant characteristics, or the duration and intensity of tDCS interventions. Nonetheless, our findings align with prior research, supporting the potential of tDCS to improve gait and balance in individuals with MS.

The positive outcomes observed in the tsDCS group may relate to the modulation of intersegmental excitability within the spinal cord, which undergoes structural and functional changes in pwMS due to demyelination and atrophy affecting both white and gray matter [36]. In healthy adults, descending and ascending spinal pathways are critical for regulating locomotor output. Previous studies have indicated that cathodal tsDCS enhances motor unit recruitment by modulating spinal pathways in healthy individuals [24]. Therefore, the positive effects observed in the tsDCS group may result from tsDCS-induced modulation of the locomotor system, possibly through effects on spinal pathways and neuronal connectivity.

Variability in the literature regarding the efficacy of tsDCS in neurological populations, particularly for motor outcomes, remains considerable. A recent systematic review [9] reported that cathodal tsDCS improved static balance and reduced resting motor thresholds and tremor frequency in individuals with primary orthostatic tremor [18], compared to sham stimulation. However, the broader range of studies included in the review showed inconsistent effects of tsDCS on motor and neurophysiological outcomes in individuals with spinal cord injury [19,20] or stroke [37], when compared to sham or other non-invasive stimulation techniques. While most studies applied tsDCS in an online paradigm (during therapy), our approach used an offline paradigm (prior to therapy). Specifically, cathodal tsDCS may enhance neural plasticity post-application, as evidenced by findings from other non-invasive stimulation techniques targeting the central nervous system [38,39]. Indeed, cathodal tsDCS has been shown to increase voluntary motor output and corticospinal transmission in healthy individuals [40], with effects persisting up to 30 min post-stimulation [41]. Hence, administering tsDCS prior to exercise may enhance motor function and mitigate fatigue.

At baseline analysis, no statistically significant differences were observed between the groups (*p* > 0.05), except for the TUG test results, which showed a significant difference (*p* = 0.040). Although the baseline values of the T25-FW test did not demonstrate a significant difference (*p* = 0.097), borderline significance was noted. The heterogeneity observed in these parameters did not affect the outcomes, as the analysis was based on pre- and post-treatment differences between the groups.

The clinical significance of these findings is highlighted by the notable improvements in walking speed and reductions in fatigue observed in both the tDCS and tsDCS groups. Given that walking impairments and fatigue are among the most debilitating symptoms for individuals with MS [42,43], our results suggest that both tDCS and tsDCS hold significant potential as therapeutic interventions to enhance mobility and alleviate fatigue. Notably, within-group improvements surpassed the minimal detectable change (MDC) and minimal clinically important difference (MCID) thresholds for tsDCS and tDCS groups. The MDC reported in the literature for individuals with MS is 2.7 s for the T25-FW test [44], with the MCID met only in the tsDCS group, which showed a 22.4% reduction, exceeding the established 20% threshold [45]. Additionally, other variables reached the MCID, in alignment with the prior literature, including 0.1–0.2 m/s for gait speed [46], over 8 points for the MSWS-12 [47], and between 0.45 and 0.88 points for FSS [48]. However, these clinically meaningful differences were not observed in the TUG and FIS tests, where MDC thresholds were set at 7.9 s [49] and between 10 and 20 points [50], respectively. Overall, these findings indicate that the observed results may hold clinical relevance for pwMS, as gait speed is closely associated with an elevated risk of falls, reduced mobility, increased disability, and fatigue, all of which significantly impact the quality of life [51].

The absence of significant differences in efficacy between tDCS and tsDCS further suggests that tsDCS could be a viable alternative for patients unable to undergo tDCS due to contraindications, such as cranial implants or post-surgical conditions. This flexibility broadens the scope of neuromodulation therapies available to pwMS. The existing literature supports the effectiveness of these techniques in modulating cortical and spinal cord excitability, leading to functional improvements. For instance, Picelli et al. [52] observed similar enhancements in motor function in stroke patients, reinforcing the potential role of combined stimulation in neurological rehabilitation. Future research should thus explore the combined application of tDCS and tsDCS in MS.

Improvements in walking function may have contributed to the physical component of fatigue, reflected in the significant reduction in fatigue severity (measured by FSS) observed in both the tDCS and tsDCS groups. These reductions were accompanied by improvements in the physical dimension of fatigue, as assessed by the FIS. Similar results have been documented in recent meta-analyses [53], which reported decreases in physical and overall fatigue following exercise interventions in pwMS. This suggests a potential link between improved motor function and reduced physical fatigue perception. However, alternative mechanisms may also contribute to these outcomes. Evidence suggests that fatigue in pwMS is more closely related to altered neural communication than to structural changes, as supported by non-invasive stimulation studies [54] that modulate neuronal excitability [53]. Although both tDCS and tsDCS can induce synaptic changes, which may influence fatigue perception beyond motor function, directly linking reduced fatigue to neurophysiological changes is challenging, as factors such as sleep disturbances and depression also affect fatigue perception [55].

One common symptom of MS is heat sensitivity [56]. Research shows that increased environmental and body temperatures can worsen feelings of fatigue. Additionally, elevated body temperature from exercise may contribute to this issue [56]. However, our study’s results indicated that the exercise program did not lead to increased fatigue. In fact, we observed a reduction in the FSS scores among the stimulation groups. We believe this positive outcome may be due to the low-intensity nature of the RAGT, which was specifically designed to aid in motor learning.

This study has several limitations. First, assessments were conducted only before and after the intervention sessions, which precludes evaluation of medium- and long-term intervention efficacy, and thus, the durability of observed effects remains uncertain. Second, although subject blinding was ensured using validated sham stimulation methods, a specific analysis to assess the success of blinding was not performed, though no indications of blinding failure were noted. Finally, the sample included participants with only two types of MS (RRMS and PPMS), potentially limiting the generalizability of the findings.

## 5. Conclusions

Both cathodal tsDCS and anodal tDCS effectively improved gait function and reduced fatigue in pwMS. The lack of differences between tsDCS and tDCS on gait and fatigue outcomes suggests that tsDCS may offer comparable therapeutic benefits, making it a suitable alternative, particularly for individuals unable to undergo tDCS due to medical contraindications. Future studies should explore the long-term effects of these interventions and examine the combined application of tsDCS and tDCS on gait-related outcomes in MS.

## Figures and Tables

**Table 1:** The content of the physiotherapy program applied to all participants for 4 weeks.

Exercises Type	Exercise Content
Robot-assisted gait training	20 min gait training: 1.5 km/h (10 min), 2.0 km/h (10 min) 20% body weight support
Strengthening exercises	Mini-squat with exercise ball support (2 sets/8 reps) Chair stand exercise (2 sets/10 reps) Step-climbing exercise (2 sets/8 reps)
Gait training	Walking in a straight line exercise (2 sets/10 m) Cross-walking exercise (2 sets/10 m) Walking exercise on ground with obstacles (2 sets/10 m)
Balance exercises	Balance exercises on soft ground Balance exercises on BOSU

**Table 2:** Comparison of the basal demographic and clinical characteristics of the groups.

	tDCS Group (n = 11) Median (Min–Max)	tsDCS Group (n = 12) Median (Min–Max)	Sham Group (n = 12) Median (Min–Max)	Comparison Between Groups
Gender (%)				
Female	8 (72.7)	8 (66.7)	9 (75.0)	0.897 ^a^
Male	3 (27.3)	4 (33.3)	3 (25.0)	
Age	49.0	49.0	48.5	
(years)	(36.0–71.0)	(30.0–75.0)	(37.0–66.0)	0.891 ^b^
Disease Duration	16.0	13.5	20.0	
(years)	(7.0–24.0)	(4.0–36.0)	(2.0–37.0)	0.510 ^b^
MS Type (%)				
RRMS	7 (63.6)	7 (58.3)	8 (66.7)	0.912 ^a^
PPMS	4 (36.4)	5 (41.7)	4 (33.3)	
EDSS Score	4.5	5.0	3.7	
	(3.5–5.5)	(3.5–6.0)	(3.0–6.0)	0.137 ^b^
T25-FW	28.6	24.1	21.0	
(s)	(14.6-39.2)	(13.5-61.2)	(15.7–26.5)	0.097 ^b^
Gait speed	0.4	0.5	0.4	
(m/s)	(0.3–0.7)	(0.2–0.8)	(0.2–0.6)	0.913 ^b^
MSWS-12	70.0	75.0	69.9	
	(52.2–83.3)	(45.0–85.0)	(30.0–91.0)	0.777 ^b^
TUG	30.0	22.8	19.2	
(s)	(10.1–38.5)	(11.3–75.1)	(9.9–30.2)	0.040 ^b^
FSS	5.0	4.8	5.3	
	(3.0–6.2)	(3.3–6.5)	(2.4–6.1)	0.578 ^b^
FIS-Phy	23.0	24.5	26.0	
	(19.0–30.0)	(14.0–30.0)	(15.0–31.0)	0.568 ^b^
FIS-Total	56.0	58.0	51.0	
	(47.0–80.0)	(39.0–76.0)	(36.0–70.0)	0.315 ^b^

tDCS, transcranial direct current stimulation; tsDCS, trans-spinal direct current stimulation; MS, Multiple Sclerosis; RRMS, Relapsing–Remitting MS; PPMS, Primary Progressive MS; EDSS, Expanded Disability Status Scale; T25-FW, Timed 25-foot Walk; MSWS-12, Multiple Sclerosis Walking Scale-12; TUG, Time Up and Go; FSS, Fatigue Severity Scale; FIS-Phy, Fatigue Impact Scale-Physical Subscale Score; FIS-Total, Fatigue Impact Scale-Total Score; ^a^ Chi-square; ^b^ Kruskal–Wallis.

**Table 3:** Intra-group and inter-group comparison before and after 4 weeks of different stimulation applications.

	tDCS Group (n = 11)			tsDCS Group (n = 12)			Sham Group (n = 12)			Inter-Group Analysis	Post Hoc Test
	Pre-Treatment Median (Min–Max)	Post-Treatment Median (Min–Max)	Intra-Group Test ^a^ *p* Value	Pre-Treatment Median (Min–Max)	Post-Treatment Median (Min–Max)	Intra-Group Test ^a^ *p* Value	Pre-Treatment Median (Min–Max)	Post-Treatment Median (Min–Max)	Intra-Group Test ^a^ *p* Value	Kruskal–Wallis Test ^b^ *p* Value	*p* (tDCS-sham) *p* (tsDCS-sham) *p* (tDCS- tsDCS)
T25-FW (s)	28.6	24.1		24.1	18.5		21.0	20.6			**0.001**
	(14.6–39.2)	(11.0–35.2)	**0.003**	(13.5–61.2)	(11.2–49.0)	**0.002**	(15.7–26.5)	(14.2–26.0)	0.084	**0.000**	**0.000**
											1.000
Gait speed (m/s)	0.4	0.5		0.5	0.6		0.4	0.4			**0.009**
	(0.3–0.7)	(0.3–1.0)	**0.003**	(0.2–0.8)	(0.2–0.9)	**0.002**	(0.2–0.6)	(0.2–0.6)	0.141	**0.001**	**0.002**
											1.000
MSWS-12	70.0	53.3		75.0	54.1		69.9	73.3			**0.008**
	(52.2–83.3)	(50.0–76.6)	**0.003**	(45.0–85.0)	(36.6–76.6)	**0.002**	(30.0–91.0)	(31.6–90.0)	0.893	**0.000**	**0.000**
											0.316
TUG (s)	30.0	25.0		22.8	15.7		19.2	18.3			**0.007**
	(10.1–38.5)	(8.8–35.2)	**0.003**	(11.3–75.1)	(8.6–54.3)	**0.002**	(9.9–30.2)	(8.8–32.1)	0.875	**0.000**	**0.000**
											0.566
FSS	5.0	4.1		4.8	4.2		5.3	5.5			**0.032**
	(3.0–6.2)	(3.1–6.3)	**0.022**	(3.3–6.5)	(2.2–5.9)	**0.007**	(2.4–6.1)	(2.1–7.0)	0.219	**0.003**	**0.004**
											1.000
FIS-Phy	23.0	19.0		24.5	18.5		26.0	22.5			1.000
	(19.0–30.0)	(17.0–27.0)	**0.021**	(14.0–30.0)	(12.0–26.0)	**0.003**	(15.0–31.0)	(16.0–32.0)	0.324	**0.049**	**0.047**
											0.350
FIS-Total	56.0 (47.0–80.0)	56.0 (47.0–76.0)	0.170	58.0 (39.0–76.0)	51.0 (36.0–70.0)	**0.041**	51.0 (36.0–70.0)	50.0 (28.0–74.0)	0.449	0.411	

DCS, transcranial direct current stimulation; tsDCS, trans-spinal direct current stimulation; T25-FW, Timed 25-foot Walk; MSWS-12, Multiple Sclerosis Walking Scale-12; TUG, Time Up and Go; FSS, Fatigue Severity Scale; FIS-Phy, Fatigue Impact Scale-Physical Subscale Score; FIS-Total, Fatigue Impact Scale-Total Score; ^a^ Wilcoxon test; ^b^ Kruskal–Wallis test was calculated using delta values (pre–post differences within each group); bold numbers indicate significant statistical differences (*p* < 0.05).

## Data Availability

The original contributions presented in this study are included in the article. Further inquiries can be directed to the corresponding author.
